# Construction of a Non-Mutually Exclusive Decision Tree for Medication Recommendation of Chronic Heart Failure

**DOI:** 10.3389/fphar.2021.758573

**Published:** 2022-02-23

**Authors:** Yongyi Bai, Haishen Yao, Xuehan Jiang, Suyan Bian, Jinghui Zhou, Xingzhi Sun, Gang Hu, Lan Sun, Guotong Xie, Kunlun He

**Affiliations:** ^1^ Department of Cardiology, The Second Medical Center and National Clinical Research Center for Geriatric Diseases, Chinese PLA General Hospital, Beijing, China; ^2^ Beijing Key Laboratory of Precision Medicine for Chronic Heart Failure, Chinese PLA General Hospital, Beijing, China; ^3^ Ping An Health Technology, Beijing, China; ^4^ Institute of Materia Medica, Chinese Academy of Medical Science and Peking Union Medical College, Beijing, China; ^5^ Research Center of Medical Big Data, The Medical Innovation Research Division, Chinese PLA General Hospital, Beijing, China

**Keywords:** decision tree, medication recommendation, clinical decision support system (CDSS), chronic heart failure, treatment, machine learning

## Abstract

**Objective:** Although guidelines have recommended standardized drug treatment for heart failure (HF), there are still many challenges in making the correct clinical decisions due to the complicated clinical situations of HF patients. Each patient would satisfy several recommendations, meaning the decision tree of HF treatment should be nonmutually exclusive, and the same patient would be allocated to several leaf nodes in the decision tree. In the current study, we aim to propose a way to ensemble a nonmutually exclusive decision tree for recommendation system for complicated diseases, such as HF.

**Methods:** The nonmutually exclusive decision tree was constructed via knowledge rules summarized from the HF clinical guidelines. Then similar patients were defined as those who followed the same pattern of leaf node allocation according to the decision tree. The frequent medication patterns for each similar patient were mined using the Apriori algorithms, and we also carried out the outcome prognosis analyses to show the capability for the evidence-based medication recommendations of our nonmutually exclusive decision tree.

**Results:** Based on a large database that included 29,689 patients with 84,705 admissions, we tested the framework for HF treatment recommendation. In the constructed decision tree, the HF treatment recommendations were grouped into two independent parts. The first part was recommendations for new cases, and the second part was recommendations when patients had different historical medication. There are 14 leaf nodes in our decision tree, and most of the leaf nodes had a guideline adherence of around 90%. We reported the top 10 popular similar patients, which accounted for 32.84% of the whole population. In addition, the multiple outcome prognosis analyses were carried out to assess the medications for one of the subgroups of similar patients. Our results showed even for the subgroup of the same similar patients that no one medication pattern would benefit all outcomes.

**Conclusion:** In the present study, the methodology to construct a nonmutually exclusive decision tree for medication recommendations for HF and its application in CDSS was proposed. Our framework is universal for most diseases and could be generally applied in developing the CDSS for treatment.

## Introduction

Heart failure (HF) is a clinical syndrome that is a result of the abnormalities in the structure and function of the myocardium impairing cardiac output or decreasing the filling of the ventricles (Metra and Teerlink, 2017). The treatment of heart failure is guided by the stage of symptoms and signs as well as a robust literature on therapies proven to be beneficial by randomized trials (Yancy et al., 2017). Despite tangible advances in recent years, HF is still a leading cause of death worldwide (Conrad et al., 2018). As a terminal stage of patients, HF is complexed with multiple comorbidities, such as coronary heart disease, hypertension, and diabetes (Chamberlain et al., 2020), making the clinical decision process complicated. Although guidelines have recommended standardized drug treatment for HF, given the complexity of HF, there are still many challenges in making the correct clinical decisions. Artificial Intelligence-Clinical Decision Support Systems (AI-CDSSs) has the potential to assist physicians in the treatment decision process in HF.

CDSSs provide evidence for physicians in making clinical decisions, such as differential diagnosis and recommending medications (Bates et al., 2001; Office of the National Coordinator for Health Information Technology, Department of Health and Human Services, 2012). The key component for providing the evidence is finding similar patients. Patients who have similar clinical conditions are expected to suffer from similar diseases and be treated with similar medications (Downie et al., 2020). Within a similar patient group, the retrospective EHR (electronic health record) data can be used to rank all candidate medications that occurred in a similar patient group (Austin et al., 2020). The way of ranking those candidate suggestions is by calculating the conditional probability (such as the fraction of diagnoses) and the effectiveness (such as prognoses after treatment with certain medication) of the suggestions.

To maintain the clinical correctness and interpretability of the model used in CDSS, knowledge-based decision trees should be constructed. A decision tree is a model consisting of consecutive decisions, starting from the root node, and each sample would be allocated to different branches based on the condition it satisfied. Nodes without downstream branches are called the leaf node, and all samples would be classified into different leaf nodes according to the decision tree. In general, the construction of such a decision tree is based on the summary of the clinical rules for a specific disease (Zhao et al., 2020). The clinical rules are composed of the conditions and actions simultaneously, indicating the actions under certain conditions. Decision trees are constructed by integrating all clinical rules to partition patients into specific subgroups represented by the leaf nodes. The conditions and corresponding actions, suggested by the clinical guidelines, are denoted in the non-leaf nodes (Song and Lu, 2015).

The core challenge in the construction of the decision tree relies on the integration of clinical rules (Ehrhardt et al., 2021). For some diseases, such as type 2 diabetes and hypertension, the whole population should be partitioned systematically according to the clinical guidelines. Therefore, the mutually exclusive decision trees are easily constructed following the clinical guidelines. For instance, in our work in the AMIA 2019 Annual Symposium (Sun et al., 2019), the whole population of diabetes patients was grouped by the HbA1c (hemoglobin A1C) value and numbers of historical antidiabetic medications according to the clinical guidelines of type 2 diabetes. In this case, each patient only belonged to one unique group. However, in most other cases, the clinical guidelines have provided independent clinical condition rules, and there are no logical exclusiveness between different clinical rules (Keikes et al., 2021). For example, for heart failure patients, the current medication recommendations are based on the previous drugs (Ponikowski et al., 2016), and historically using one drug A is not mutually exclusive with using another drug B. Therefore, it is unrealistic to integrate those recommendations into a mutually exclusive decision tree.

It is challenging to convert clinical guidelines into a normal decision tree since those trees, where one patient can only be allocated in a unique leaf node, are designed to be mutually exclusive. To tackle this problem, we proposed a novel way to construct and leverage a nonmutually exclusive decision tree in CDSSs. The construction process was just listing all the clinical rules horizontally if they were independent. Therefore, each branch was not mutually exclusive with others in the decision tree. One patient could enter multiple branches and be allocated to several different leaf nodes simultaneously.

**FIGURE 1:**
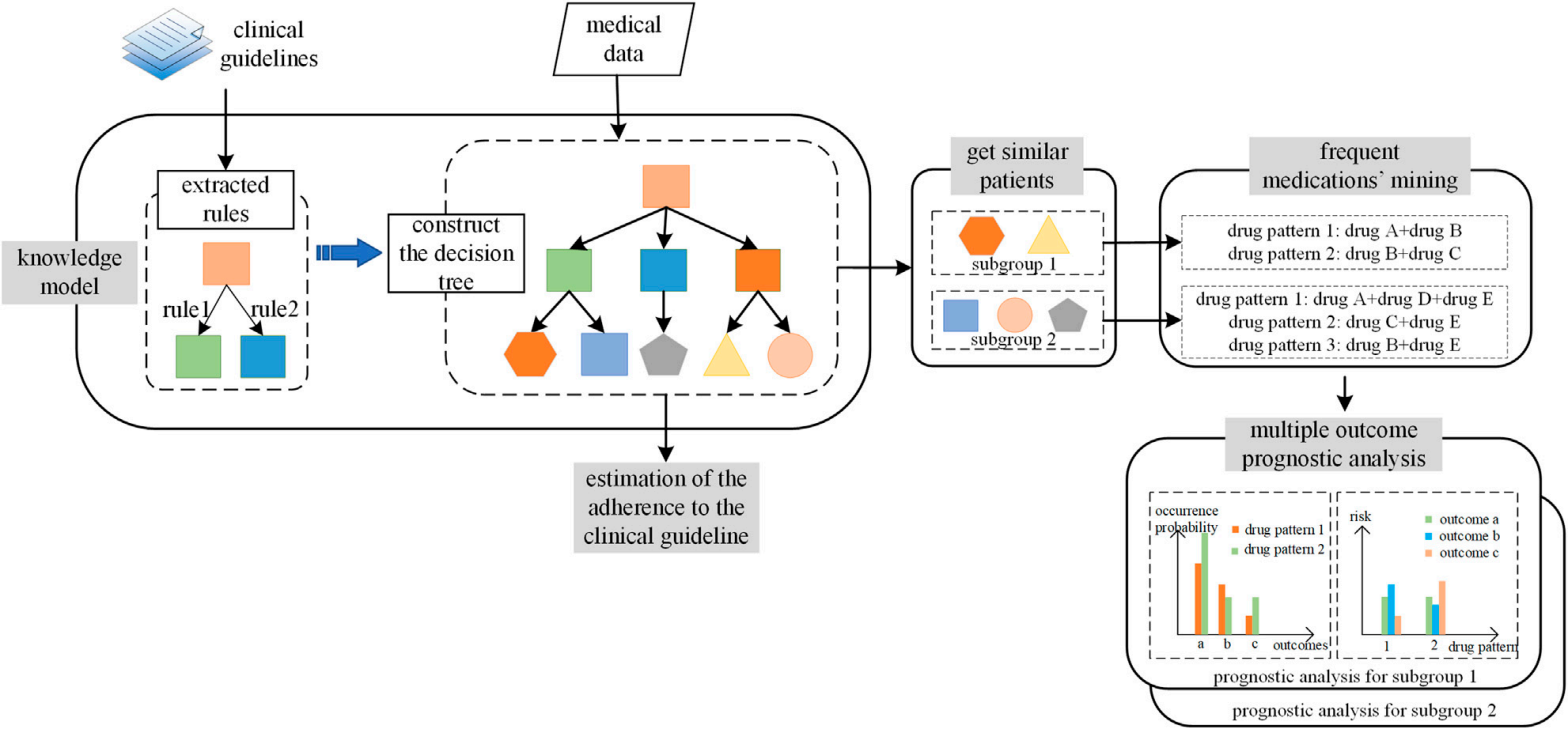
The workflow to construct and apply nonmutually exclusive decision tree. The workflow included two parts. The first part is the procedure of the construction of nonmutually exclusive decision tree, denoted as “knowledge model.” The second part is the application of the nonmutually exclusive decision tree, which included tree components: the fine group of the patients, the frequent medication patterns mining for each patients group, and the multiple outcomes prognoses analyses.

In the current study, we proposed a framework to construct the nonmutually exclusive decision tree and applied it in the HF treatment recommendation as shown in Figure 1. The three essential components for CDSS in application included the definition of similar patients, the medication patterns for each similar patient group, and evidence-based recommendation strategy. For the first component, similar patients were defined as patients following the exact same leaf node allocation patterns in the nonmutually exclusive tree. Second, to obtain the medication patterns for each similar patient group, the frequent mining algorithm was applied. Third, to provide real-world evidence, we analyzed the prognoses for each medication pattern, which were calibrated by the propensity score of a patient following a particular medication pattern. The multiple prognoses provided a multidimensional view for physicians when making decisions.

## Methods

This study was approved by the ethics committee of the Chinese PLA General Hospital.

### Datasets

The dataset we used was extracted from a heart failure database in the Chinese PLA General Hospital in Beijing, China. This database was initially constructed for all patients diagnosed with heart failure from 2008 to 2018. The database included 29,689 patients with 84,705 admissions in total. According to the classification and diagnostic criteria of heart failure in the guidelines (Ponikowski et al., 2016), all samples that satisfied the definition of HF with reduced ejection fraction (HFrEF) were included in the current study. A total of 9,414 patients with 16,063 visits were obtained. Each record consisted of demographic information, physical examinations, laboratory test results, medication history, and current medication.

It should be noted that the quality of the data was critical in providing real-world evidence. We used the data of inpatients instead of those of outpatients, considering that the coverages of many features of the data of inpatients were much higher than the data of the outpatients. Such substitution hypothesized that the medication for inpatients and outpatients of HfrEF was similar in terms of medication categories, such as ACEIs (angiotensin-converting enzyme inhibitors) or β-receptor blocker, and our CDSS also provided real-world evidence in the granularity of medication categories. The medication considered in the current study is listed in Table 1. Another hypothesis was that the relative day of the admission for one patient was insignificantly related to the medication decisions, which we had checked in the current study by regressing the medication decisions with the relative day as the condition.

**TABLE 1:** The medication considered in the current study.

Medications for HFrEF treatment	Abbreviation	ATC codes
Angiotensin-converting enzyme inhibitor	ACEI	C09AA
Angiotensin Ⅱ receptor blocker	ARB	C09CA
Angiotensin receptor neprilysin inhibitor	ARNI	C09DX04
β-receptor blocker	β	C07AB, C07AA, C07AG
Aldosterone receptor antagonist	ARA	C03DA
Diuretics	Diu.	C03CA, C03XA
Digitalis	Dig.	C01AA
Ivabradine	Iva.	C01EB

### Data Preprocessing

The preprocessing of the data of inpatients contained three steps: data standardization, data segmentation, and missing value imputations.

First, the medications used in the current study were standardized by the ATC (Anatomical Therapeutic Chemical) five-digit code as listed in Table 1.

**FIGURE 2:**
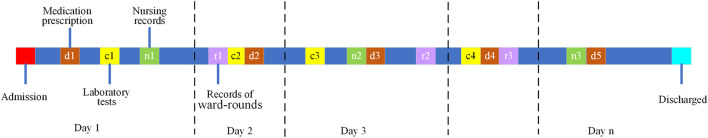
The schematic diagram of an *n*-day electronic health record (EHR) for inpatient. The data would be segmented into n fragments as indicated by the vertical dash lines, and the colorful boxes represent the different categories of information: d for the prescript medications, c for the results of the laboratory tests, *n* for the nursing records, and *r* for the records of the ward rounds.

Second, the data of the inpatients were fragmented by the day. As shown in Figure 2, the records of the inpatients with a length of stay equal to n could be converted into *n* samples. For each sample, the history was referred to as the activities that happened in the previous day. It should be noted that by multivariable logistic regression to the prescriptions, the day indexes in the segmented daily records were independent of the medications in each day. A total of 132,158 samples were obtained after data fragmentation.

Third, a two-step strategy of missing value imputation was utilized in the current study. First, the missing values were imputed forward or backward for the same admission. Then the rest of the missing values were imputed with means for continuous variables and medoids for discrete variables. The coverage of each variable before and after the first step imputation is listed in Table2.

**TABLE 2:** The coverage of each variable before and after the forward and backward imputation.

Variable name	Coverage before the imputation^a^	Coverage after the imputation
Heart rate	96.72%	98.88%
Serum potassium	95.72%	99.83%
Serum sodium	95.08%	99.77%
B-type natriuretic peptide (BNP)	94.12%	—^b^
Creatinine	93.80%	99.62%
Systolic blood pressure	59.80%	97.83%
Urine volume	57.03%	84.76%
Cardiac function level	37.66%	84.76%
Ejection fraction	26.19%	59.81%

Note. a Only the first step imputation: forward and backward imputation of the same patients.

b We did not impute BNP, since it was used to estimate one outcome (BNP_improved).

### Construction of Knowledge-Based Non-mutually Exclusive Decision Tree

To construct the knowledge model, clinical recommendations were first summarized and extracted from the clinical guidelines. Then the clinical recommendations were organized in the form of a decision tree with each branch representing one recommendation in the clinical guidelines. The decision-tree-like knowledge model was constructed and reviewed by multiple clinical experts.

As shown in Table 3, the clinical recommendations for HfrEF were extracted from the 2016 ESC guidelines for the diagnosis and treatment of HF (Ponikowski et al., 2016). According to the clinical guidelines, the treatment of HfrEF was divided into three phases: the new cases without historical HF treatment, cases after the initial treatment, and end-stage heart failure cases. The last phase was excluded since there was no medication-related treatment. For each phase, the clinical conditions and the recommended treatments were listed horizontally. For the new cases, the prescriptions were made based on whether there were symptoms of congestion. Besides, for the ones who had congestion symptoms, patients were further divided according to the existence of hyponatremia and the tendency of renal function damage. For cases after the initial treatment, the medication decisions were determined by the previous treatment and the current symptoms.

**TABLE 3:** Extracted clinical rules for the treatment of HFrEF according to the clinical guidelines.

	Clinical situation			Recommended treatment
	Historical medication	Others		
New cases	—	Have symptoms and/or signs of congestion	There was no hyponatremia and no tendency of renal function damage	Loop diuretics|thiazide Diu. + ACEI/ARB + β
			There is hyponatremia or tendency of renal function damage	Tolvaptan + ACEI/ARB +β
		No symptoms and/or signs of congestion		ACEI/ARB +β
Cases after initial treatment	Containing diuretics	Congestion symptoms improved		The original diuretic regimen was maintained for further observation
		Congestion symptoms did not improve		Add tolvaptan
	Containing ACEI/ARB +β	Improvement of heart failure symptoms		Maintain the original treatment plan and continue to observe
		The symptoms of heart failure did not improve	eGFR ≥ 30 ml min^−1^·1.73m^−2^ and serum potassium <5.0 mmol/L	Addition of aldosterone receptor antagonist
			NYHA cardiac function class –I–III and blood pressure can tolerate ACEI/ARB (systolic blood pressure ≥95 mmHg)	Replacing ACEI/ARB with ARNI
			β has reached the target dose or the maximum tolerated dose, sinus heart rate ≥70 beats/min and LVEF ≤ 35%	Addition of Iva.
			According to CRT/ICD indications	CRT/ICD
			The combination of multiple treatments still has symptoms	Add Dig.
End-stage heart failure	—	—	—	Heart transplantation|palliative care|left ventricular assist device

Note. *Medication referred to Table 2.

Abbreviations: eGFR, estimated glomerular filtration rate; NYHA, the New York Heart Association Functional Classification; LVEF, left ventricular ejection fraction; CRT, cardiac resynchronous therapy/ICD (cardiac resynchronous therapy).

**FIGURE 3:**
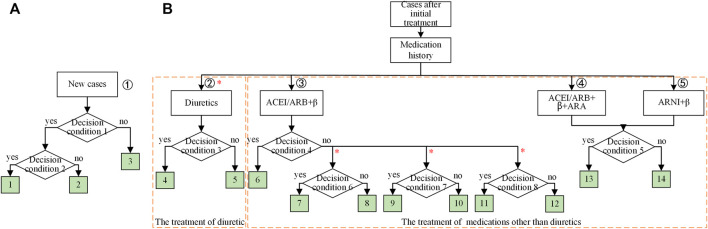
The integrated knowledge-based decision trees for heart failure (HF) with reduced ejection fraction (HFrEF): **(A)** the decision tree for new cases; **(B)** the decision tree for cases with initial treatment of HFrEF. The nonmutually exclusive branches were labeled with the red *. For **(B)**, the medications were grouped into two independent parts as indicated by the dashed boxes. The leaf nodes were colored in green, indicating the medications for each branch. The details for decision condition 1 was the existence of congestion symptom; for decision condition 2, the existence of hyponatremia or the tendency of renal function impairment; for decision condition 3, the whether the congestion symptoms got improved; for decision condition 4, whether the heart failure symptoms got improved; for decision condition 5, whether the LVEF ≤40% and no symptom improvement with a combination of multiple medications; for decision condition 6, whether the eGFR ≥30 ml min^−1^,·1.73 m^−2^, and the blood potassium <5.0 mmol/L; for decision condition 7, whether the NYHA was between II and III and the tolerance of ACEI/ARB (the systolic blood pressure ≥95 mmHg); for decision condition 8, whether the LVEF ≤35%, the sinus heart rate ≥70 beats/min, and β-receptor blocker reached the target dose (or the maximum tolerated dose). Abbreviations: eGFR, estimated glomerular filtration rate; NYHA, the New York Heart Association Functional Classification; LVEF, left ventricular ejection fraction.

To construct the decision tree, the extracted clinical rules were integrated in the following ways. If the clinical conditions were independent, the clinical rules were organized horizontally; otherwise, they were integrated following the logical hierarchy. The constructed decision tree for the new cases and the cases after initial treatment were shown, respectively, in Figures 3A, B. The leaf node represented the treatment strategies as listed in Table 3. Note that the leaf nodes indexed by 4, 8, 10, 12, and 14 stood for the same treatment as previous ones.

It should be noted that in Figure 3B, the decision tree had four branches, and the leftmost one (marked with a red star) was independent of the other three. Thus, patients allocated in the leftmost branch would not be mutually exclusive with the other three. The clinical records would be allocated to leaf node 4 and leaf node 6 at the same time. All the nonmutually exclusive branches were marked with a star in Figure 3B.

### Similar patients and medication recommendation

#### Similar patients

After the knowledge tree was constructed, the clinical records would be allocated to a set of leaf nodes. In the current study, we proposed defining similar patients as those who followed the same pattern of leaf node allocation. For example, patients allocated to leaf node 4 and leaf node 6 were treated as the same patient group. Note that only the exact leaf allocated patterns were counted in the fine grouping of the patients.

#### Frequent Medications’ Mining for Each Subgroup and Each Non-Mutually Exclusive Part in the Decision Tree

To provide real-world clinical evidence, for each patient subgroup, the frequent drug patterns were mined using the Apriori algorithms (Ding et al., 2008). The Apriori algorithm was a powerful tool to mine the frequent patterns for transaction data. By iteratively adding candidate frequent items into the *k*th frequent itemsets, the *k+1*st frequent itemsets were generated. Then those generated candidates were excluded once they did not satisfy the frequency threshold. The remaining *k+1*st frequent itemsets were used for the next round of generation.

It should be noted that the nonmutually exclusive feature led to the independence of the frequent medication patterns between nonmutually exclusive parts in the decision tree. As shown in Figure 3B, the prescription for a particular patient would be the combination of the diuretics with other medicines related to heart functions, such as ACEI and β-receptor blocker. Therefore, the frequent medication patterns would be mined, respectively, for diuretics and heart function-related drugs.

#### Prognosis’s Analyses

##### Multiple Outcomes

The real-world evidence for each subgroup was provided based on the prognosis analyses. The real-world evidence played a critical role for leaf nodes with variable choice of medications. Therefore, multiple outcomes were considered in the current study to assist the physician to make decisions even for the same patient subgroup.

To assess each treatment effect of HfrEF, six outcomes were considered in the current study. They are hyponatremia, hypernatremia, hypokalemia, hyperkalemia, acute kidney injury, and the reduction of B-type natriuretic peptide. They could be grouped into the following three categories:

(1) Electrolyte disturbance

In the treatment of HfrEF, electrolyte disturbances mainly referred to the blood potassium and the blood sodium. The high or low blood potassium and the blood sodium were all considered as electrolyte disturbances. Hyponatremia is defined as blood sodium <135 mmol/L, while hypernatremia is defined as blood sodium >145 mmol/L. The lower limit for blood potassium was 3.5 mmol/L, and the upper limit was 7.0 mmol/L. Any values out of the range were defined as hypokalemia or hyperkalemia accordingly.

(2) Acute kidney injury

Heart failure and chronic kidney disease often coexist, and the existence of renal insufficiency could worsen the prognosis of HF (Bock and Gottlieb, 2010). The following standard was used to judge the occurrence of acute kidney injury (a severe case for renal insufficiency) (Khwaja, 2012): the creatinine rises 26.5 µmol/L within 48 h or 1.5 times of the baseline within 7 days (increase >50%), and urine output of <0.5 ml kg^−1^ h^−1^ (time >6 h).

(3) B-type natriuretic peptide

The B-type natriuretic peptide (BNP) was one of the most common heart failure biomarkers used in screening, diagnosis, severity assessment, and prognosis of heart failure. Also, it was an indicator of the risk of cardiovascular events in patients with heart failure after discharge. The lower the BNP values, the better the clinical conditions the patient had. We chose a 20% reduction of BNP as the indicator of improvement of clinical conditions for HF, denoted as BNP_ improved^17^. The 20% increase in BNP was labeled as the deterioration of HF conditions.

##### Medication Assessments Using Propensity Score

As aforementioned, the treatment using diuretic and other HF drugs were independent; the prognosis analyses were also carried out separately for diuretic and other HF drugs. For each group of similar patients with each nonmutually exclusive part, suppose there were *n* candidate medications, and there would be 6*n* prognosis analyses since there were six outcomes considered in the current study.

To calibrate the bias introduced by patients using the specific medication, the propensity score of the medications was included in the regression to the outcomes. To be specific, for the prognosis analysis with treatment T, other variables X, and the outcome Y, we first regressed T on X and obtained the propensity score of a patient using treatment T. Then the propensity score, as well as the variable X, was used to the regression of the outcome Y. The formula for the second regression was:

$$Y=\sigma \left(\beta_{PS}\mathrm{PS}+B^{T}X+\beta_{T}T+b\right)$$

where σ is the sigmoid function, which transforms any value to a probability between 0 and 1. The equation is:

$$\sigma \left(x\right)=\frac{1}{1+e^{-x}}$$

The PS was the propensity score of a patient choosing the treatment T, and $\beta_{PS}$ , $\beta_{T}$ , and B were the learnable parameters for the propensity score, the treatment variable, and other variables, respectively. According to the potential outcome framework (Rubin, 2005), after being calibrated with the propensity score of the selection bias of the treatment, the estimation of correlation between the treatment and the outcome would be more reasonable. Finally, $\beta_{T}$ was used to reflect the propensity of treatment T to the occurrence of the outcome Y. A positive $\beta_{T}$ indicated a promoted effect to the outcome.

## Results

### Loading all Clinical Records to the Knowledge-Based Decision Tree for Heart Failure With Reduced Ejection Fraction

#### Historical Medication Patterns’ Mining and Expansion of the Knowledge-Based Decision Tree

**FIGURE 4:**
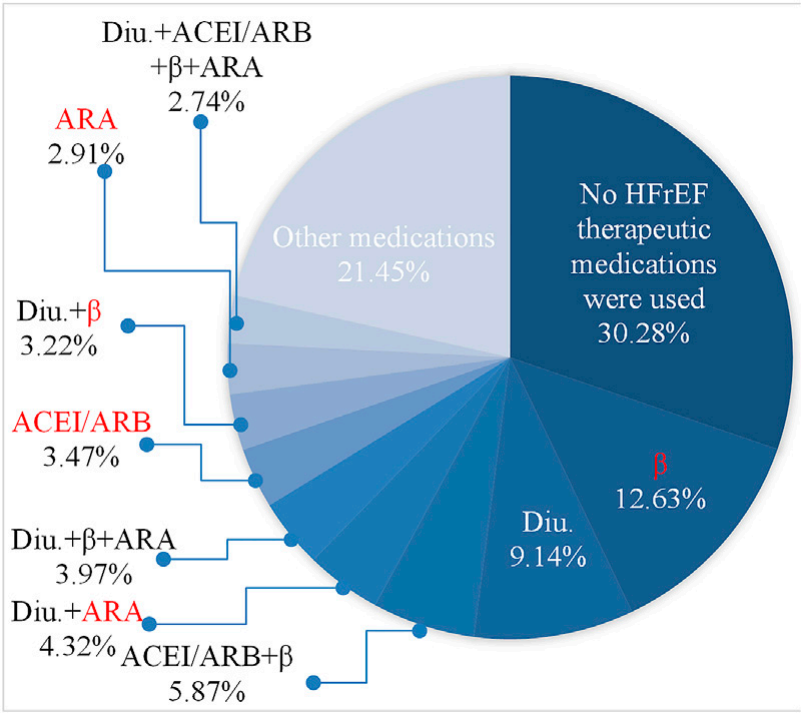
Distribution of the mined frequent historical medications for all HFrEF clinical records (132,158 in total). The top 10 medications were listed with the name and the proportions. Please see Table 1 for the abbreviations of the medicines. The medicines labeled in red were not considered as historical medication strategies in the decision trees for HFrEF.

According to the HfrEF knowledge decision tree, the first decision point was the history of medication of the patient. To understand the distribution of medication history, we first mined the history medication using the Apriori algorithms (Agrawal and Srikant, 1994) as introduced in the method section and identified the frequent sets of medications with a support threshold of 0.05%. The frequent set is shown in Figure 4. Only the top 10 medication strategies had their name listed in Figure 4 for the sake of clarity. The top 10 medication strategies accounted for 78.55% of the total sample of 132,158. The most popular medication strategy was no medication, which accounted for 30.28%. Those samples would be loaded into the decision tree for new cases (see Figure 3A), while the remaining 69.72% would be loaded into the decision tree shown in Figure 3B.

Among all samples loaded for Figure 3B, 47,028 records were successfully loaded into the tree, which accounted for 35.58% (47,028/132,158) of the candidate samples. The main reasons came from the missing historical medication strategies in the knowledge-based decision tree. The other reason for records failed to be loaded into Figure 3B was the missing value for clinical conditions, such as the ejection fraction. It should be noted that the records were loaded before the second imputation step.

**FIGURE 5:**
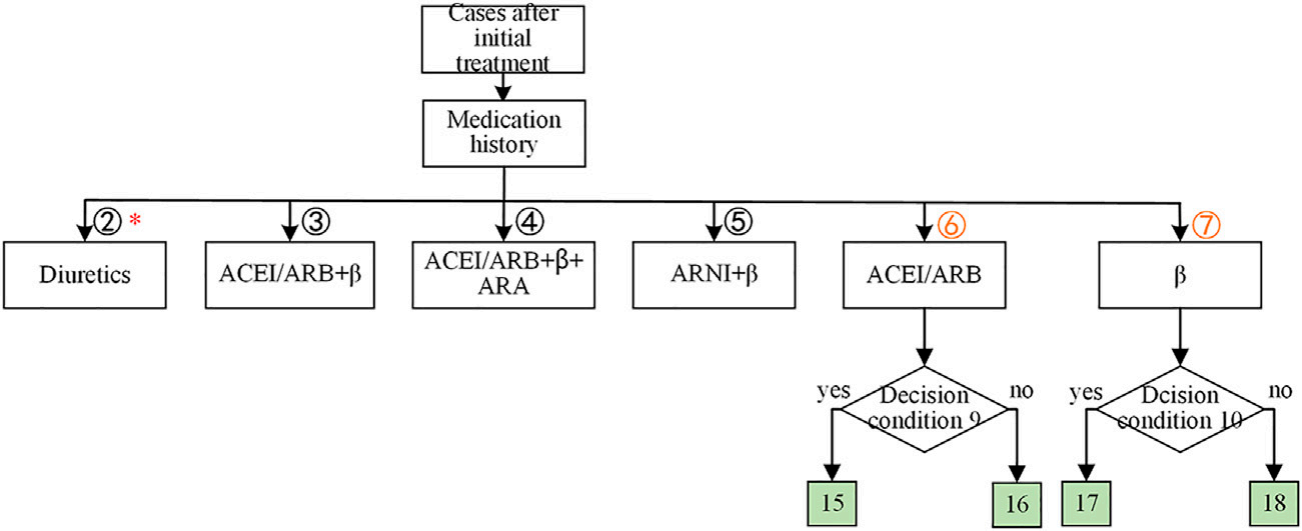
The expansion of the knowledge-based decision tree for cases with initial treatment of HFrEF. The nonmutually exclusive branches were labeled with red *. The leaf nodes were colored in green, indicating the medications for each branch. The decision condition 9 was whether the systolic blood pressure >90 mmHg or the heart rate >60 beats/min. The decision condition 10 was whether the systolic blood pressure >90 mmHg or the heart rate >60 beats/min.

As shown in Figure 4, historically, only β-receptor blocker was used for heart function accounting for 15.85% of the records, which consisted of using β-receptor blocker (12.63%) only and using β-receptor blocker with diuretics (3.22%). The same reason for the other historical medication strategies was denoted in red in Figure 4. Considering that HF patients only had β-receptor blocker (15.85%), or ACEI/ARB (angiotensin II receptor blocker) (3.47%) were common in clinical practice, the knowledge decision tree for the cases after initial treatment got expanded as shown in Figure 5. As shown in Figure 5, there were two expanded branches: branch (6) and branch (7). Branch (6) was ACEI/ARB, and branch (7) was a β-receptor blocker. The corresponding decision condition 9 was defined as systolic blood pressure >90 mmHg or heart rate >60 beats/min, and decision condition 10 was systolic blood pressure >90 mmHg or heart rate >60 beats/min. Leaf node 15 represented adding β-receptor blocker based on ACEI/ARB medication, leaf node 16 represented maintaining the original medication ACEI/ARB, leaf node 17 represented adding ACEI/ARB based on β-receptor blocker, and leaf node 18 indicated maintaining the original medication β-receptor blocker.

#### Ambiguous clinical conditions

Since the existence of some subjective or hardly recorded clinical conditions, it may be difficult to run through the decision tree. Thus, we proposed their alternative estimations. For example, the decision condition 1/3/4 (the existence of congestion symptoms/the improvement of congestion symptoms/the improvement of heart failure symptoms) was difficult to assess directly by using our data. For the decision condition ¾, changes in the BNP values were used instead (Nassif et al., 2019). More specifically, if the BNP value decreased by more than 20%, the symptoms were defined as improved, and vice versa. For decision condition 1, there was no substitute for judgment, so we did not analyze the corresponding branch.

#### Estimation of the Adherence to the Clinical Guideline for Each Leaf Node

After loading the records to the decision tree, we estimated the adherence of each clinical node to the clinical guideline. By adherence, we meant the medication patterns were not in conflict with the clinical guidelines. To estimate the adherence, the frequent medication patterns were mined for each leaf node, and the top ones were used to be compared with the guideline-recommended medications. The top five frequent medications and the corresponding guideline adherence for each leaf node are listed in Table 4. By the time the dataset was generated, there was no usage of new drugs, such as tolvaptan, ARNI (angiotensin receptor neprilysin inhibitor), and ivabradine. Therefore, the leaf nodes involving the new drugs were excluded from the following analyses, such as leaf node 4. Most of the leaf nodes had a guideline adherence of around 90%, except leaf nodes 7 and 13.

**TABLE 4:** The frequent set of leaf node medication for HFrEF (top 5).

Leaf node no.	The medications recommended by clinical guidelines	The top 5 medication strategies (separated by comma)	Guideline compliance^a^
4	Diu.	**Diu.**, **Diu. +ARA**, **Diu. +β+ ARA**, **Diu. +β**, **Diu. +β+ ARA + Dig**.	84.39%
6	(Diu.) + ACEI/ARB+β	**Diu. +ACEI/ARB+β+ ARA**, **ACEI/ARB+β**, **Diu. +ACEI/ARB+β +ARA + Dig.**, **ACEI/ARB+β+ ARA**, **Diu. +ACEI/ARB+β**,	93.57%
7	(Diu.) + ACEI/ARB+β+ARA	ACEI/ARB+β, **Diu. +ACEI/ARB+β+ARA + Dig.**, **Diu. +ACEI/ARB+β+ ARA**, **ACEI/ARB+β+ ARA**, **ACEI/ARB+β+ ARA + Dig**.	44.22%
8	(Diu.) + ACEI/ARB+β	**ACEI/ARB+β**, **Diu. + ACEI/ARB+β+ ARA + Dig.**, **Diu. + ACEI/ARB+β+ ARA**, **Diu. +ACEI/ARB+β**, **ACEI/ARB+β+ ARA**	94.62%
10	(Diu.) + ACEI/ARB+β	**ACEI/ARB+β**, **Diu. + ACEI/ARB+β+ ARA + Dig., Diu. + ACEI/ARB+β+ ARA**, **Diu. + ACEI/ARB+β+ ARA**, **Diu. +ACEI/ARB+β**	94.62%
13	(Diu.) + ACEI/ARB +β+ARA + Dig. or (Diu.) + ARNI+β+ Dig.	**Diu. +ACEI/ARB+β+ ARA + Dig.**, Diu. +ACEI/ARB+β+ ARA, ACEI/ARB+β+ARA, **ACEI/ARB+β+ ARA + Dig.**, Diu. +β+ ARA + Dig.	54.43%
14	(Diu.) + ACEI/ARB +β+ ARA or (Diu.) + ARNI+β	**Diu. +ACEI/ARB+β+ ARA + Dig.**, **Diu. +ACEI/ARB+β+ ARA**, **ACEI/ARB+β+ ARA**, **ACEI/ARB+β+ ARA + Dig.**, Diu. +β+ ARA	93.47%

Note. a Guideline compliance was calculated as the proportion of medications that were including recommended by the guidelines. The bold font indicated the medications that obeyed the guidelines.

### Similar Patients for Non-Mutually Exclusive Decision Tree

Once the clinical records were loaded into the decision tree, the patients were clustered into different subgroups according to our definition of similar patients. The leaf node patterns and sample size of the top 10 subgroups are listed in Table 5. The most popular pattern was allocated to leaf node 5 only, which accounted for 18.54% of all running through clinical examples. Subgroup c was the most popular pattern that contained multiple leaf nodes.

**TABLE 5:** Leaf node patterns and sample size for the top 10 subgroups.

Subgroups no.	The leaf nodes	Number	Ratio (%)
A	5	24,508	18.54
B	4	6,952	5.26
C	7,12	3,007	2.28
D	5, 7, 10, 12, 14	2,278	1.72
E	7, 10, 12	2,057	1.56
F	4, 6	1,061	0.80
G	5, 7, 12, 14	1,035	0.78
H	6	967	0.73
i	7, 10, 12, 14	821	0.62
j	7, 12, 14	724	0.55

### Prognostic Analyses for Similar Patients and Medication Recommendation Strategy

**FIGURE 6:**
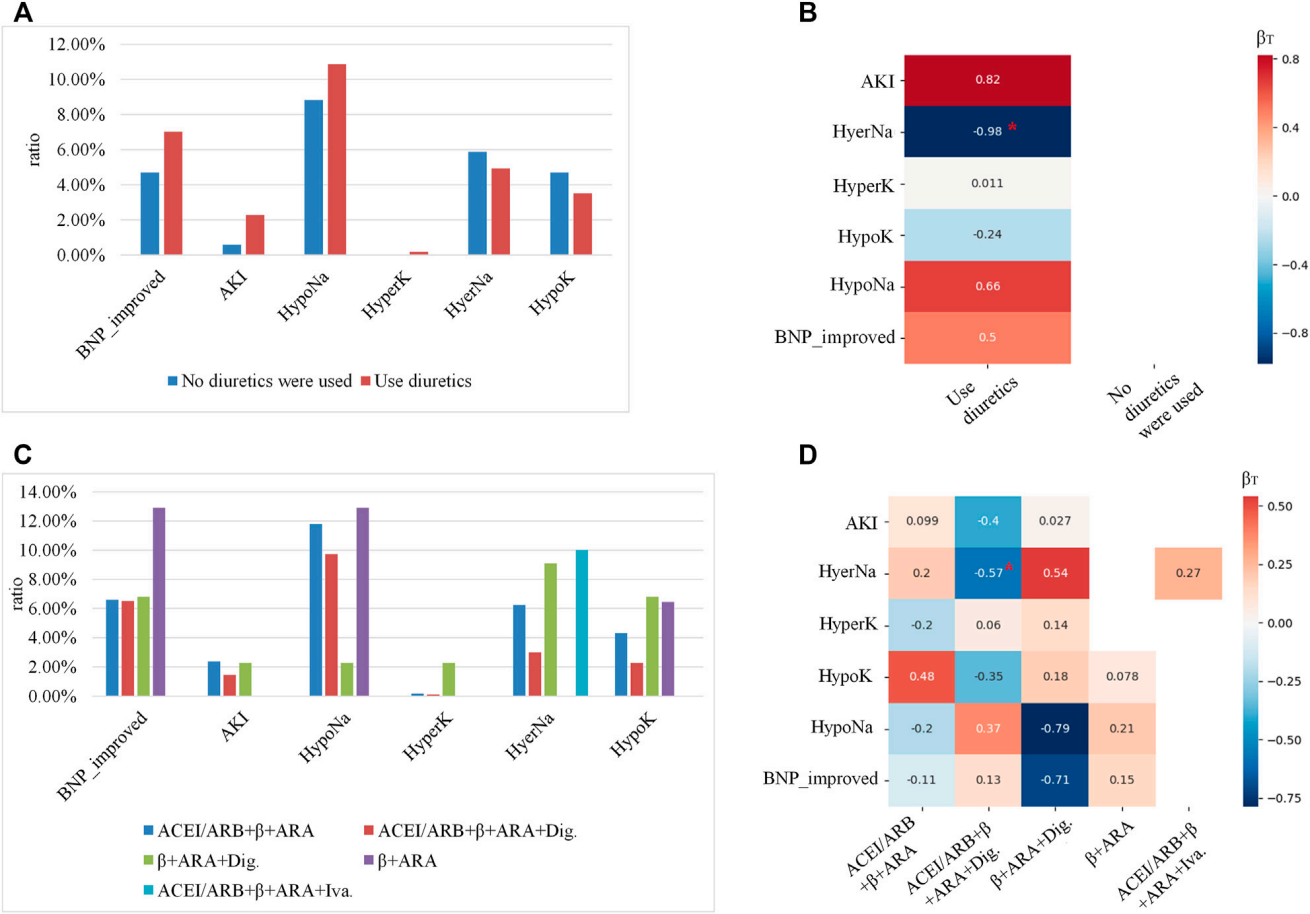
The prognosis’s analyses for the subgroup (allocated to leaf nodes 5, 7, 10, 12, and 14): **(A,C)** showed the occurrence ratios of each outcome for different medications, where the x-axis indicted the different outcomes, the y-axis indicated the occurrence ratio, and the color denoted different medications. **(B,D)** were 2D heatmaps indicating the calibrated coefficients of each medication for different outcomes, where the x-axis showed the medications, the y-axis indicated the outcomes, and the color was in proportion to the coefficients. Red colors denote the risk factors for the outcomes, and the blue ones denote the protective factors. The coefficients with p-value <0.05 were labeled with red *.

As introduced in *Prognosis analyses*, all combinations for the top five medications and the six outcomes were analyzed for each leaf node. As a running example, here we showed the prognosis analysis for subgroup d. For subgroup d, patients were allocated to five leaf nodes simultaneously. As the prescription of the diuretics and heart functional-related drugs was independent according to the clinical guidelines, the prognostic analyses were carried out separately. Shown in Figures 6A, B are the prognostic analyses on whether to use diuretics, while Figures 6C, D are the analyses for heart function-related medications. The occurrence ratios for each medication are shown in Figures 6A, C, while the calibrated coefficients of each medication for different outcomes are indicated in Figures 6B, D.

As shown in Figure 6A, using diuretics for subgroup c had a higher ratio of improving BNP level, and also the risk of AKI and HypoNa. According to Figure 6B, using diuretics only would significantly lower the risk of occurring HyperNa. As shown in Figure 6C, the medication of β+ ARA (aldosterone receptor antagonist) benefitted the improvement of the BNP level the most, but would also increase the risk of HypoNa. As indicated by Figure 6D, using ACEI/ARB+β+ARA + Dig. Would significantly lower the risk of occurring HyperNa.

## Discussions

In the present study, we proposed a framework to construct a nonmutually exclusive decision tree and to combine the tree with real-world data for a treatment recommendation. Based on this framework, we successfully built the CDSS for chronic heart failure treatment with a large real-world inpatient dataset. In addition, the multiple outcome prognosis analyses were carried out to assess the medications for the subgroup of each similar patient, which facilitated the physicians in making decisions in a patient-specific way.

Although many achievements have been made in improving the model precision for medication recommendations (Liu et al., 2017; Shang et al., 2019; Chowdhury and Turin, 2020), it is still necessary to maintain the interpretability and ensure consistency with clinical knowledge in the real application of CDSS. Therefore, there is a trend in combining clinical guidelines and retrospective I data. Wei Zhao et al. proposed to construct a decision tree with clinical rules extracted from the clinical guidelines by the Gini impurity calculated by using the real data (Zhao et al., 2020). Sun et al. (2019) proposed to integrate the real-world evidence calculated using data with the knowledge-based decision trees. In the present study, we found data helped in expanding the knowledge-based decision tree for cases with the initial treatments for HFrEF. Also, the mining of the frequent medication patterns enriched the knowledge-based tree, especially when the prognosis analyses showed benefit for some outcomes.

The decision tree is composed of the clinical conditions mentioned in the clinical guidelines and the candidate medications in the leaf nodes. In the mutually exclusive settings, following the clinical conditions on the decision trees, one patient is allocated to a unique leaf node. Those required a systematic partition of the whole population, which was infeasible for most cases, especially for complicated diseases such as chronic heart failure.

As proposed in the current manuscript, the construction of the nonmutually exclusive decision tree simply organized the independent clinical rules horizontally. The similar patients were defined as the same patterns of leaf nodes allocated, and the preparation of the real-world evidence for each subgroup of similar patients relied on the two-step linear regression for the nonmutually exclusive parts separately. In summary, the three key components for nonmutually exclusive decision trees, the construction process, the similar patients, and the real-world evidence were nondisease specific; thus, the methodology would be a general solution for all diseases without a systemic partition of the whole population.

The limitation of the current work was that we only used single-center data and had not yet tested the construct CDSS with an external dataset. Besides, the effect of the substitution of the data of outpatients for the data of the inpatients was not carefully evaluated in the application of CDSS.

The novelty of our work relied on how to utilize such a nonmutually exclusive decision tree in CDSS. First, one key concept in CDSS was identifying similar patients, which were defined as patients assigned to the same set of leaf nodes. Second, to provide real-world evidence, we separated different types of medications and recommended independent medications for each similar patient group. To make precise medication recommendation for each patient, the prognoses evidence for each treatment should be calibrated by the propensity score of a particular treatment. In practice, the calibration included two steps. First, the propensity for patients in a particular patient group to choose one medication pattern were evaluated by regression to the medication patterns. Second, the effect of each medication pattern to the outcome should be calibrated by considering the propensity of medication chosen when regressed to the outcome. Therefore, our nonmutually exclusive decision tree would provide risks of different outcomes for each medication pattern of each patient group, which would assist physicians to make medication decisions for a specific patient.

In conclusion, in the present study, the methodology to construct a nonmutually exclusive decision tree for medication recommendations for HFrEF and its application in CDSS was proposed. Our framework is universal for most diseases and could be generally applied for developing the CDSS for treatment. This provides a promising solution for diseases that are infeasible to obtain a mutually exclusive decision tree for treatment.

## Data Availability

The raw data supporting the conclusion of this article will be made available by the authors, without undue reservation.
